# Supplementary material for: Insights into the biodegradation of pentachlorobiphenyl by Microbacterium paraoxydans: proteomic and metabolomic studies
Source: Front Microbiol. 2024 Jun 12;15:1389805. doi: 10.3389/fmicb.2024.1389805 (PMC11203399; doi:10.3389/fmicb.2024.1389805)
Supplement: Supplementary file 1 [file Data_Sheet_1.docx]

**Insights into the Biodegradation of Pentachlorobiphenyl by *Microbacterium paraoxydans*: Proteomic and Metabolomic Studies**

Lei Ji ^a, 1, *^, Xiaoyu Chang ^a, 1^, Leilei Wang ^a^, Xiaowen Fu ^a^, Wenkai Lai ^c^, Liwen Zheng ^a^, Qi Li ^a^, Yingna Xing ^a^, Zhongfeng Yang ^a^, Yuyao Guan ^b, *^, Fenglong Yang ^c^^, *^

^a^ Shandong Provincial Key Laboratory of Applied Microbiology, Ecology Institute, Qilu University of Technology (Shandong Academy of Sciences), Jinan 250103, China

^b^ Department of Pharmacy, Shandong Provincial Third Hospital, Cheeloo College of Medicine, Shandong University, Jinan 250031, China

^c^ Fujian Key Laboratory of Medical Bioinformatics, Department of Bioinformatics, School of Medical Technology and Engineering, Fujian Medical University, Fuzhou 350122, China

* Corresponding authors.

E-mail addresses: jilei.1010@163.com (L. J), guanxiaoyao0815@163.com (Y. Guan), yangfenglong@fjmu.edu.cn (F. Yang).

^1^ To be considered as joint first authors.

**SUPPLEMENTARY MATERIALS**

**Figure S1** The growth curve of *M. paraoxydans* in LB (Luria-Bertani) medium (A), MSM (Minimal Salt Medium) and MSM supplemented with PCB101 (B).


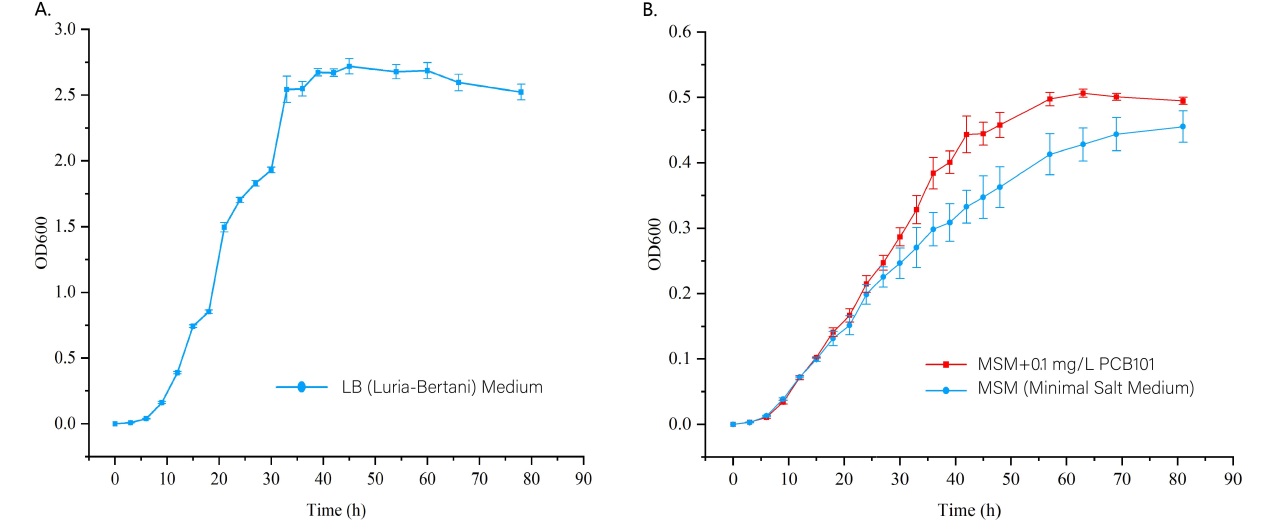


**Figure S2 Protein secretory and export pathways were enriched among the up-regulated proteins induced by PentaCB.** In the proteomics analysis of *M. paraoxydans* incubated with PentaCB, we detected Signal peptidase I (SPase I), crucial for translocated preprotein release from the cytoplasm to the periplasm, and TatA, a component of the twin-arginine translocation (Tat) system. This suggests that enzymes responsible for PentaCB degradation were distributed both in the cytoplasm and on the outer surface of the cytoplasmic membrane. The image was sourced from the KEGG database, and the up-regulated relevant proteins induced by PentaCB are outlined in red boxes.


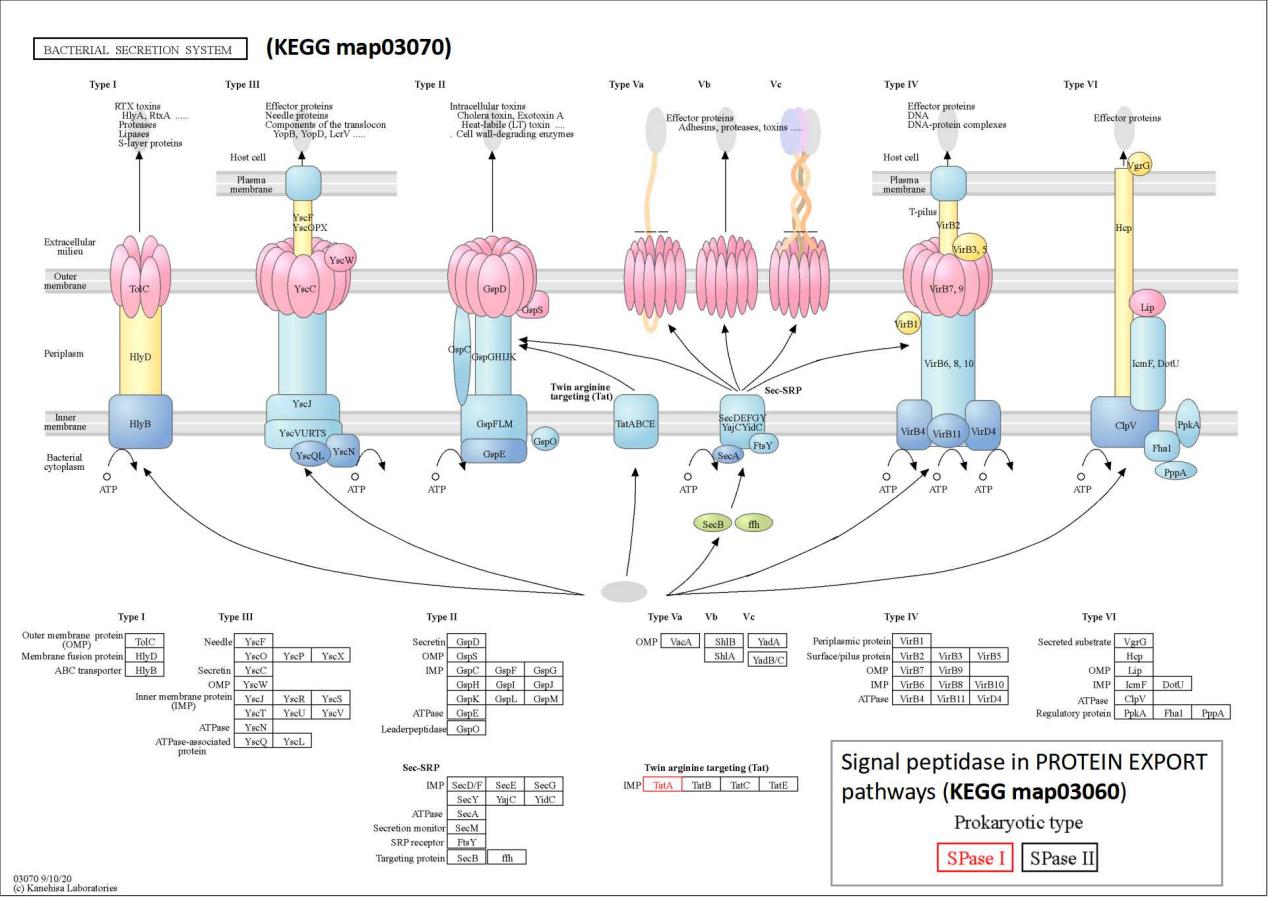


**Table S1 LB medium**

| Component | Concentration (g·L^-1^) |
| --- | --- |
| Peptone | 10.0 |
| Yeast extract | 5.0 |
| NaCl | 10.0 |

**Table S2 Induction medium MSM**

| Component | Concentration (g·L^-1^) |
| --- | --- |
| Peptone | 2 |
| (NH_4_)_2_SO_4_ | 1.0 |
| NaCl | 0.2 |
| KH_2_PO_4_ | 0.5 |
| K_2_HPO_4_ | 0.5 |
| MgSO_4_ | 0.2 |
| CaCl_2_ | 0.1 |

**Table S3 PBS buffer**

| Component | Concentration (g·L^-1^) |
| --- | --- |
| KH_2_PO_4_ | 0.24 |
| Na_2_HPO_4_ | 1.44 |
| NaCl | 8.0 |
| KCl | 0.2 |

# Appendix S1 Bacterial Screening Protocol for bacteria with high efficiency in degrading PentaCB

**S1.1 Sample dilution and inoculation**

Extract the leachate from POPs (persistent organic pollutants)-contaminated soil and dilute it to five concentration gradients of 10^-1^, 10^-2^, 10^-3^, 10^-4^ and 10^-5^. The diluted bacterial suspension was then evenly spread onto MSM (Table S2) agar plates containing biphenyl and incubated at 30°C for 1-3 days and the growth of the colonies was observed.

**S1.2 Strain separation and purification**

Colonies of bacteria that exhibited rapid growth and a typical morphology were selected and then purified through three consecutive streak plating procedures. A single colony was then inoculated into an inorganic salts liquid medium (Table S2) and cultured at 30°C with shaking at 150 rpm for three days. Subsequently, 1.5 mL of the culture was mixed with 0.5 mL of glycerol, homogenized, and stored long-term at -80°C.

**S1.3 Screening of enzyme-producing strains**

The above-obtained strains were inoculated separately onto an inorganic salts liquid medium MSM (Table S2) containing 0.1 mg/L concentrations of PCB101 (2, 2', 4, 5, 5'-pentachlorobiphenyl). The cultures were then incubated at 30°C with shaking at 150 rpm for 72 hours. Turbidity of the bacterial cultures was observed, and the absorbance at 600 nm of the bacterial suspensions was measured. The cultures were collected and centrifuged (8000 rpm, 4°C) for 5 min. The bacterial sediment was washed with the phosphate buffer solution (PBS, pH7.0) three times. The wet weight of the collected cells was measured by an analytical balance. Then the collected cells were resuspended in 10mM potassium phosphate buffer (pH 7.0, 4mM DDT) and sonicated at 320 W for 17 min with sonication time 2s and quench time 2s (Nanjing Shunma, SM-650D). The volume of the buffer added was 20 times the mass of the cells. After ultrasonic treatment, the supernatant was collected as the PentaCB-degrading enzyme and its activity of PentaCB degradation was analyzed. Based on these criteria, enzyme-producing strains were selected, identified by 16S rDNA full-length sequencing and deposited in the China General Microbiological Culture Collection Center (CGMCC).

**Appendix S2 TMT Proteomic Quantification and Functional Analysis**

**S2.1 Total Protein Extraction**

Cells were lysed using liquid nitrogen and then ultrasonication individually. The lysate was centrifugated and the supernatant was collected. The protein concentration was determined using the Bradford protein assay. Extracts were reduced and then alkylated with iodoacetic acid. Samples were precipitated with acetone, collected, and washed with cold acetone. The precipitate was dissolved in buffer, following tests of protein concentration.

**S2.2 Peptide Preparation and TMT Labeling**

Supernatants containing 0.1 mg of protein were subjected to Trypsin Gold digestion (enzyme-to-substrate ratio 1:50). Peptides were desalted, dried, and labeled with TMT6/10-plex reagents. After 1 h incubation, the reaction was stopped with ammonium hydroxide. Differentially labeled peptides were combined and desalted. A common reference sample was created from pooling aliquots from each sample.

**S2.3 HPLC Fractionation and LC-MS/MS Analysis**

TMT-labeled peptides were fractionated using a C18 column on a Rigol L3000 HPLC with a gradient elution. Eluates were collected and combined into 15 fractions. The fractions were dried under vacuum and reconstituted in 0.1% formic acid (FA). 2 μg peptide samples were introduced into a column, where peptide separation was achieved using a gradient elution method. EASY-nLC 1200 UHPLC system coupled with an Orbitrap Q Exactive HF-X mass spectrometer were used for shotgun proteomics sequencing.

**S2.4 Identification and Quantitation of Protein**

Spectra were searched against the *M. paraoxydans* UniProt database using Proteome Discoverer 2.2. Search parameters included mass tolerances, fixed and variable modifications, and miscleavage sites. Protein identification required FDR < 1% on both peptide and protein levels. Proteins with similar peptides, indistinguishable by MS/MS analysis, were grouped separately as protein groups. TMT quantification was performed using Reporter Quantification. The Mann-Whitney Test was used in statistical analysis.

**S2.5 Functional Analysis of Protein and DEP**

GO and IPR analyses were performed using the interproscan-5 program against the non-redundant protein database (Pfam, PRINTS, ProDom, SMART, ProSiteProfiles, and PANTHER). Protein family and pathway analysis utilized the COG and KEGG databases. Potential interacting partners were predicted using the STRING-db server (http://string.embl.de/), which contains both known and predicted protein-protein interactions (Franceschini et al., 2013). Enrichment analysis of GO, IPR, and KEGG was performed using an enrichment pipeline (Huang et al., 2008). Differentially expressed proteins (DEP) were identified using significant ratios (p < 0.05 and |log_2_FC| > 1.2).

**Appendix S3 UPLC-QTOF-MS Metabolomics Quantification and Analysis**

**S3.1 Metabolites Extraction and GC-MS/MS Analysis**

Completely dry the samples using a vacuum concentrator without applying heat. Then, add 60 μL of methoxyamination hydrochloride (20 mg/mL in pyridine) and incubate the mixture at 80°C for 30 min. 80 μL BSTFA reagent (1% TMCS, v/v) was added to the sample aliquots followed by incubation at 70 °C for 1.5 h. GC system coupled with a Pegasus HT TOF-MS was employed for analysis.

**S3.2 Data Analysis**

Data processing included peak extraction, baseline correction, peak alignment, deconvolution analysis, peak identification, and area integration were performed using LECO Chroma TOF 4.3X software and the LECO-Fiehn Rtx5 database. Metabolite identification relied on both mass spectrum matching and retention index matching.

**REFERENCES**

Franceschini, A., Szklarczyk, D., Frankild, S., Kuhn, M., Simonovic, M., Roth, A., Lin, J., Minguez, P., Bork, P., Mering, C., Jensen, L.J., 2013. STRING v9.1: protein-protein interaction networks, with increased coverage and integration. Nucleic Acids Res. 808-815.

Huang, D.W., Sherman, B.T., Lempicki, R.A., 2008. Bioinformatics enrichment tools: paths toward the comprehensive functional analysis of large gene lists. Nucleic Acids Res. 37(1), 1-13.
